# Experimental determination of the stabbing intensity in an intracranial stabbing death

**DOI:** 10.1007/s00414-025-03622-y

**Published:** 2025-10-21

**Authors:** Johann Zwirner, Matthias Vollmer, Mario Scholze, Sven Anders-Lohner, Michael Morlock, Benjamin Ondruschka

**Affiliations:** 1 Institute of Legal Medicine, University Medical Center Hamburg-Eppendorf, Hamburg, Germany; 2 Department of Oral Sciences, University of Otago, Dunedin, New Zealand; 3 Institute of Biomechanics, TUHH Hamburg University of Technology, Hamburg, Germany; 4 Institute of Materials Science and Engineering, Chemnitz University of Technology, Chemnitz, Germany

**Keywords:** Degree of force, Force magnitude, Forensic biomechanics, Lethal head impact, Stabbing

## Abstract

An evaluation of the stabbing intensity including the degree of force necessary to cause a particular injury is a common task for forensic pathologists in court. Biomechanical analyses are essential for collecting objective data, serving as a baseline comparison among the highly individual circumstances of each case. However, previous investigations have utilized instruments only resembling the murder weapons as well as tissues from individuals other than the victim, including animal tissues or substitutes, which limits their applicability to forensic casework. In this study, a homicidal head stab case is presented, in which the blade penetrated over its full width, crossing the midline and injuring the brainstem and the contralateral hemisphere. A skull sample from the victim’s contralateral side corresponding to the injured region was retrieved during the autopsy. For the stabbing experiments, a pendulum setup incorporating the original blade was employed. Three consecutive stabs were executed on the bone sample of the victim obtained at autopsy. Additionally, two other skull samples from different cadavers were each subjected to a single stab. The stabbings were performed at varying bone thicknesses (3–8 mm) and momenta (3.1–13.4 Ns) to account for mild, moderate, and strong impacts. High impact velocities resulted in either a blade entry across its full width, resembling the homicide case, or a multi-fragmental destruction of the bone. Mild and moderate impacts were insufficient to achieve full-thickness penetration of the skull. When stabbings were performed on a considerably thicker skull sample than that involved in the homicide case, only the blade tip penetrated the bone without achieving full-thickness perforation. By utilizing tissue from the victim and the real weapon for biomechanical analysis of the stabbing intensity including the minimum degree of force and momenta in homicidal stab cases, this experimental setup closely mimics the conditions of the actual case. Forensic investigators should proactively recommend such biomechanical analyses and secure appropriate tissue samples during autopsy to obtain objective experimental data relevant to legal questions.

## Introduction

Homicides involving penetrating head injuries with knives are extremely rare events in Western countries [1, 2]. In court, forensic pathologists are frequently asked to comment on the degree of force associated with a particular injury, especially when sharp force was applied. A high degree of force may indicate the assailant’s intent to cause harm, while a low degree of force could be interpreted as an accidental scenario. However, assessing the degree of force is challenging due to the complex interplay of numerous influencing factors, including blade shape, blade sharpness, impact velocity, the anatomical region impacted, and the movement of the victim [3–6]. Only biomechanical experiments designed to determine the degree of force can provide forensic investigators with objective evidence on this matter.

Previous investigations of head stabs with knives are limited, with only one study analyzing the depth of blade penetration across three different thrust momenta [6]. These experiments were motivated by a homicide case in which a person was killed by stabs from a jackknife. While the cause of death was attributed to a stab to the heart, the victim also suffered an intracranial stab with full blade penetration. Based on 80 individual tests conducted on five different cadavers, excluding the victim, and using a knife equivalent to the murder weapon, the study established a minimum momentum necessary to achieve a penetration depth consistent with that observed in the homicide case.

However, biomechanical experiments aimed at quantifying the degree of applied force have faced criticism. Critics argue that the highly controlled settings of these experiments may not account for all intrinsic and extrinsic factors influencing injuries, such as the sex and age of the deceased and skull thickness [3]. Theoretically, the corresponding contralateral skull bone should closely resemble the affected skull region, as all intrinsic factors are kept stable. Thus, the corresponding contralateral head region might represent the ‘ideal control’, which has also been the case for brain tissue in immunohistochemical studies [7–9].

In this report, we present a homicidal head stab case followed by biomechanical experiments aimed at determining the stabbing intensity including the minimum degree of force and the momenta to penetrate the skull. To the best of our knowledge, this is the very first study to utilize tissue from the victim taken from the contralateral head region as well as the original blade of the weapon for this purpose.

### Case report

**Fig. 1:**
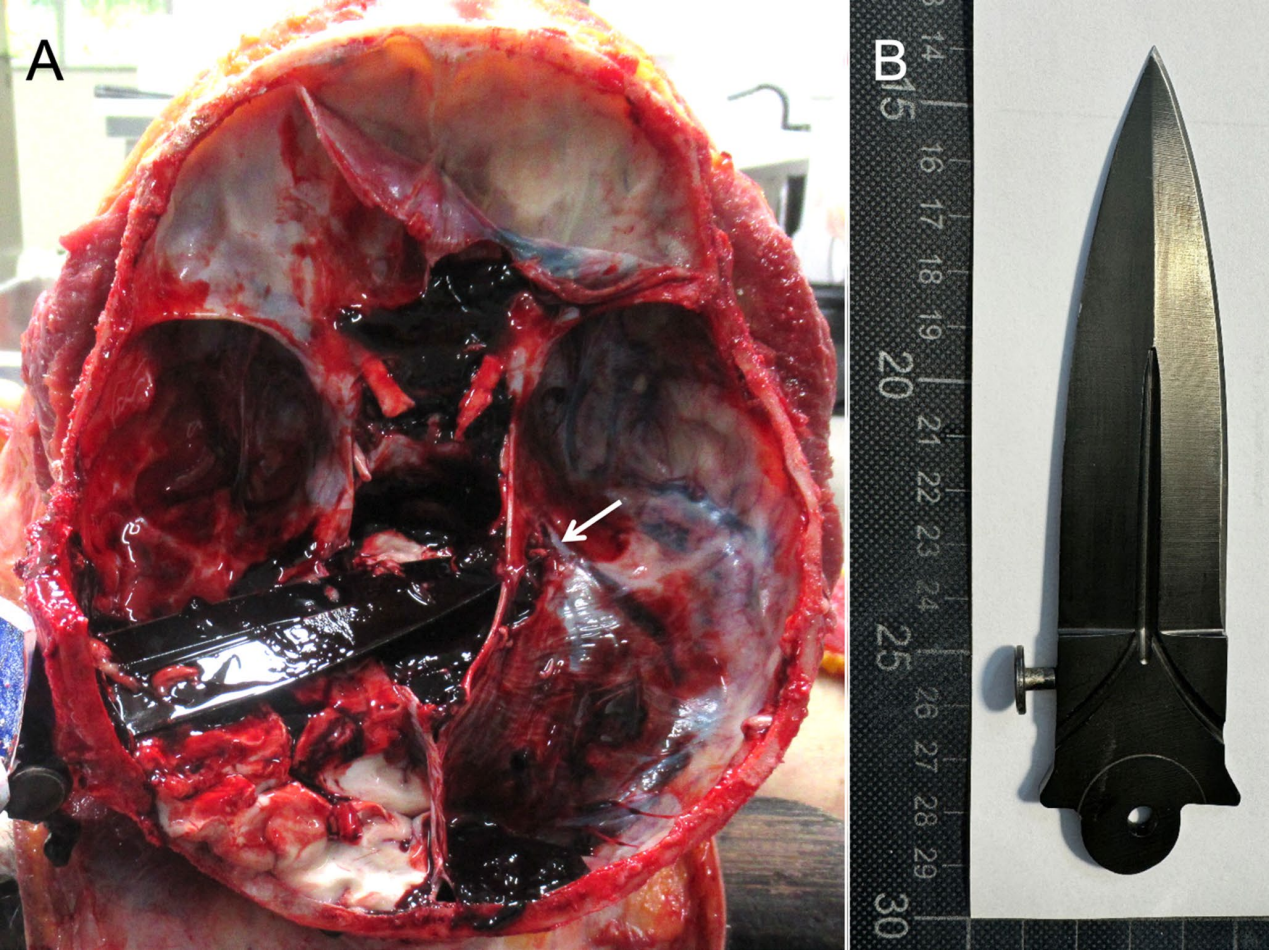
Images of the autopsy and the murder weapon are shown. **(A)** The knife is stuck in the left temporo-parietal region over the full blade width. The tip crossed the midline and led to a laceration in the right cerebellar tentorium (white arrow). Subdural bleeding zones are visible. **(B)** The blade of the original knife with a one-sided grind is shown after securing evidence and cleaning

An adult man sustained a single intracranial stab in his left temporo-parietal region in a fight. He was transferred to a hospital under cardiopulmonary resuscitation, where he was soon declared brain dead. He died immediately after cessation of the artificial ventilation. Autopsy revealed that the blade of the knife was firmly stuck in the skull over its full width and blade length (Fig. 1A). The temporo-parietal skull thickness within the affected region was determined to be 3–4 mm in post-mortem computed tomography (CT) images carried out prior to autopsy. The blade crossed the midline and cut the posterior part of the brainstem. It’s tip ended in the right cerebellar tentorium in the area of the superior petrosal sinus. Signs of minor blunt force traumata on the oral mucosa, the left eyebrow, the bridge of the nose, the extensor side of the left index finger, the right knee joint, the left tibia and the left forearm were consistent with the reported fight prior to death. A triangular skull piece of the contralateral side including the region corresponding to the stabbing defect was retained during autopsy with attached periosteum. A biomechanical analysis of the degree of force was recommended to the public prosecutor’s office by our forensic institute. The public prosecutor’s office followed the forensic recommendation and ordered the biomechanical analysis to determine the degree of force that was necessary to perforate the skull. To allow for the best possible simulation of the case, the original knife (Fig. 1B) could be used for the biomechanical experiments after DNA and dactyloscopy evidence had been secured.

## Materials and methods

### Experimental setup

**Fig. 2:**
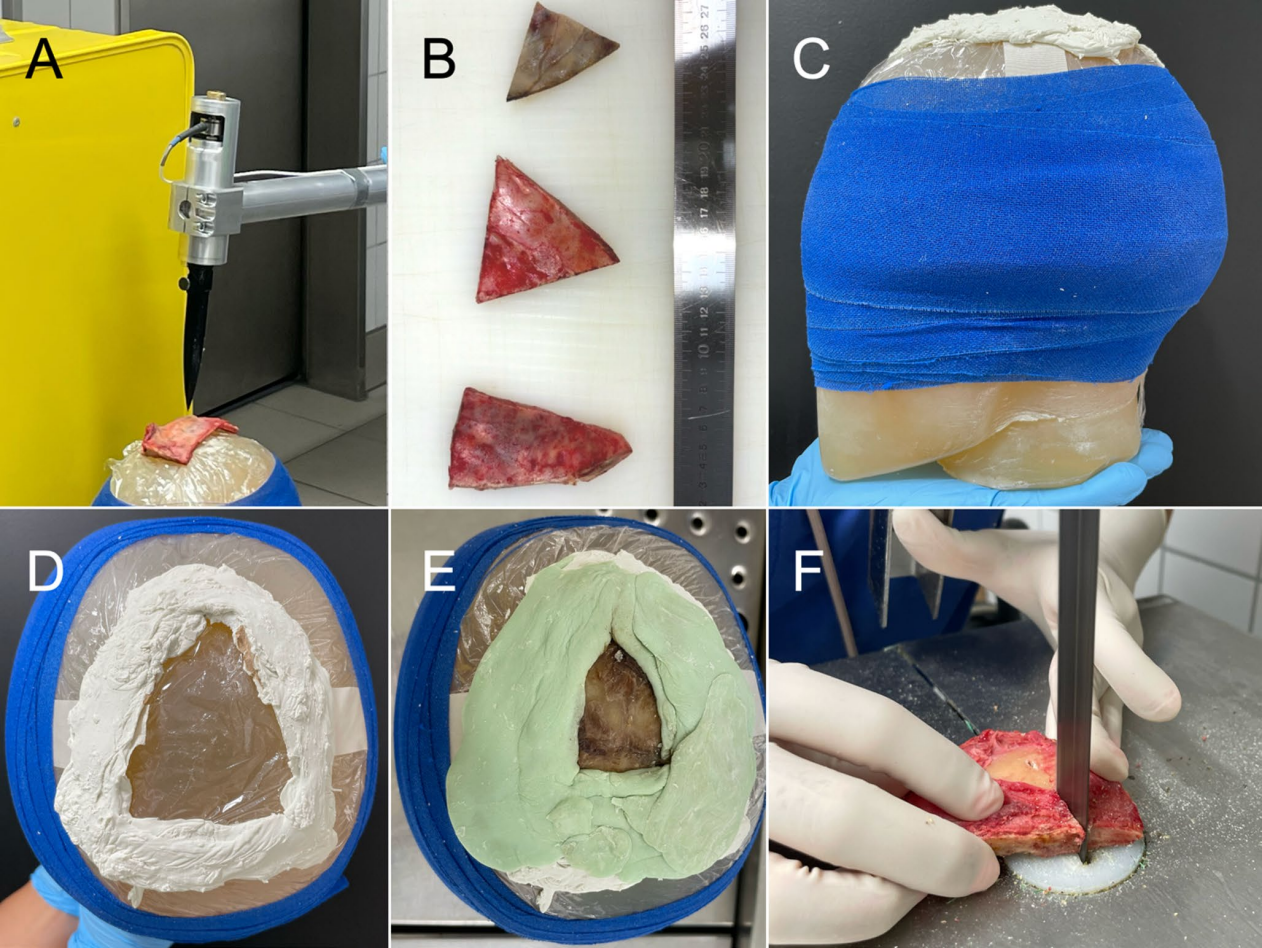
Representative images of the materials and methods of the tests are depicted. **(A)** A pendulum setup with the original blade affixed on top was used. **(B)** The three used bone samples are depicted. **(C)** For testing, the bones were placed on top of a ballistic soap head. **(D)** Bone cement underneath the planned position of the sample retrieved from the victim is shown. **(E)** The sample of the victim was then fixated with bone cement around the edges. **(F)** The experimental bone defects were sawed along their length to determine the penetration depth of the blade

For the stabbing simulation, the original blade was affixed to a pendulum measuring 1 m in length and 3 kg in weight (Fig. 2A). The pendulum was height-adjustable so that the blade could pierce the measuring surface perpendicularly. The base of the blade was connected to a 5 kN load cell (type 8431 − 6005; Burster, Hamburg, Germany). The signal from the load cell was amplified with a measuring amplifier (LCV-U10; ATP Messtechnik, Ettenheim, Germany). This analog output force signal was digitally converted with the analog-to-digital converter (USB-6002; National Instruments, Austin, USA) and read into a laptop via a USB interface. A data acquisition program was created using the LabView 2013 development environment, which records and displays the force signal and saves it as a force-time curve. A total of 6000 values were recorded per measurement sampled at 10 kHz. The recorded signal length was 600 ms in each case. Each measurement was initiated by a trigger set to 15 N. The triangular skull samples with side lengths between 4 and 9 cm for the mechanical tests originated from three different individuals: the macroscopically intact temporo-parietal region of the victim contralateral to the injury, the temporal region of another cadaver, and the occipital region of a third individual (Fig. 2B). Firstly, samples from other than the victim were included to increase the number of tests and thus the meaningfulness of the conducted experiments. Secondly, the particular sample locations including a markedly thicker sample from the occipital region were selected with the intention to demonstrate the influence of the sample thickness on the load resistance of the sample. The bone sample from the victim was stored at −20 °C between the autopsy and six hours prior to the mechanical tests to prevent degradation. The bone samples from the other two individuals were retrieved during routine autopsies on the day of testing and stored at 4 °C until the tests were conducted. The bone samples were placed on a ballistic soap head according to VPAM AND-PrM (Schubert, Magdeburg), which was covered with transparent film in the impact area and a gauze bandage away from the impact area to prevent debris from flying toward the investigators in the event of fracture (Fig. 2C). The ballistic soap head was positioned on a standard autopsy table. The skull sample of the victim was secured using bone cement (Palacos; Heraeus Medical, Hanau, Germany) underneath and around the edges of the sample (Fig. 2D, E) to prevent dislocation during and after the tests, as three consecutive tests were performed. The two remaining skull samples were placed on the ballistic soap head without applying bone cement, as only one test was conducted for each. The skull sample of test 5 was secured with tape to prevent bone fragments from flying through the testing room in case of small-sized fragmentation. The impact energy was deemed sufficiently high so that sample slippage during the test - potentially affecting the measured maximum forces - was negligible. The bone sample of the victim was initially impacted without manual acceleration by releasing the pendulum at a 90° angle to the impact plane (test 1). It was then attempted to impacted the samples with moderate (test 2) and high (test 3) manual acceleration by the investigator operating the testing device. The skull samples from the other two cadavers including the one of similar thickness to the sample of the victim (test 4) and one considerably thicker than the one of the victim (test 5) were impacted similar to test 3 (attempt to impact at high acceleration). The tests were videorecorded using a mobile phone camera with a frame rate of 240 fps (iPhone 15; Apple, Cupertino, USA) manually held in fixed position in-plane with the impact setup to measure the impact velocities and, thereby, validate the different impact accelerations. Motion velocities were approximated from the video recordings using a reference scale and an open-source motion tracking software (Tracker 6.2.0; Open Source Physics, www.physlets.org/tracker). The impact velocities were analysed by tracking the speeds at multiple points along the knife, and the average velocity was calculated for each test. The corresponding velocity value just before the impact or skull penetration was defined as the impact velocity for each test.

To determine the thicknesses of the bone samples within the impact area, the skull samples were sawed along the length of the defect created during the experiments (Fig. 2F) and measured with a ruler. Additionally, CT scans of all three bone samples were performed after the mechanical tests, allowing for the assessment of defect depth in cases where the bone was not fully perforated.

## Results

Of the three stabs delivered to the bone of the victim, only the third stab, which involved high manual acceleration, resulted in penetration of the knife across the full width of the blade, as observed in the corresponding homicide case. In contrast, high manual acceleration of the pendulum caused a twofold fragmentation of a skull sample of similar thickness from another individual. A skull sample from a different individual, which was twice the thickness of the injured region in the homicide case, withstood a considerably higher impact force without allowing the knife tip to penetrate fully through the three-layered skull.

**Fig. 3:**
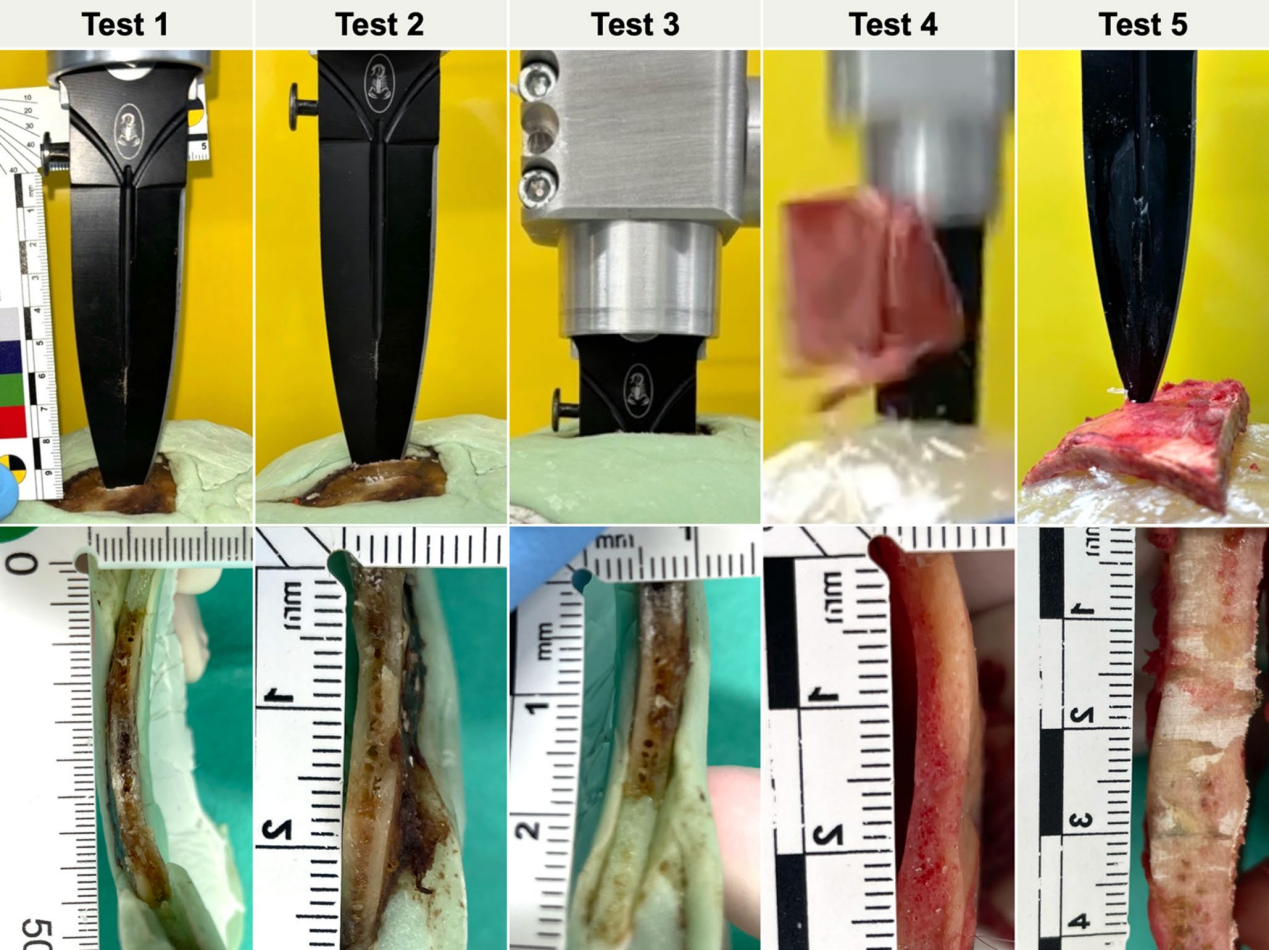
Images of the penetration depth of the knife blade of each test are shown in the upper row. The lower row depicts the bone thicknesses in the impacted areas, which were visible after sawing the defects along their length. Please note that the image of test 4 appears blurry due to the limited frame rate of the camera

A summary of the results, including relevant background information, is presented in Table 1. Images depicting the penetration depth of the knife blade for each test, as well as the bone thicknesses of the impacted areas, are shown in Fig. 3.

**Table 1:** An overview of the five consecutive tests using the original blade

	Test 1	Test 2	Test 3	Test 4	Test 5
Origin	Victim	Victim	Victim	Individual 1	Individual 2
Manual acceleration	None	Moderate	High	High	High
Impact velocity [m/s]	10 (37 km/h)	12 (41 km/h)	15 (55 km/h)	22 (79 km/h)	17 (61 km/h)
Skull thickness [mm]	3–4	3–3.5	3–4	3–4	6–8
F_max_ [N]	435	550	431	387	1368
Momentum [Ns]	3.1	3.5	10.1	8.6	13.4
Impact duration [ms]	10.2	11.0	41.1	32.6	22.0
Penetration depth [mm]	10 (blade tip)	20 (blade tip)	100 (full blade width)	Bone fragmentation	5 (blade tip)

## Discussion

Experimental approaches to determine the degree of force in stabbing incidents have been reported for various instruments, including knives [4–6, 10–12], screwdrivers [11, 13], and sickles [14]. However, previous studies have predominantly utilized instruments similar to those used in real cases, along with tissues from cadavers other than the victim [12], animals [11, 15], or proxies [4, 13]. Consequently, a primary critique of biomechanical experiments aimed at determining the degree of force is that they are not directly transferable to forensic casework [3].

To the best of our knowledge, this study is the first to determine the degree of force in a homicidal knife stabbing case using tissue from the victim as well as the original blade of the murder weapon. Consequently, this approach directly addresses the critical perspective that biomechanical experiments may not be applicable to forensic casework [3]. By utilizing tissue from the contralateral side of the injured skull region, factors such as age, sex, and potential diseases affecting bone quality can be accurately represented. For the neurocranium, three-point bending tests of human skull samples in both semi-static and dynamic settings have shown that the maximum forces before sample breakage are similar between the left and right temporal regions [16, 17]. Furthermore, assuming that the skull is reasonably symmetrical and free from previous injuries, essential factors such as skull thickness, vascular imprints [18], and anatomical regions can be best replicated and even verified using widely available non-invasive techniques such as CT.

The experiments revealed that a minimum momentum of 10.1 Ns was required to achieve full-width penetration of the blade into the skull of the victim using the original knife. This momentum was attained with high manual acceleration of the pendulum, resulting in an impact velocity of 55 km/h. In contrast, either no manual acceleration or moderate manual acceleration did not achieve full-width blade penetration; both conditions were insufficient to replicate the circumstances of the homicide case satisfactorily. A study on the biomechanics of knife stab attacks including 17 volunteers measured the velocity, momentum and energy when stabbing on an artificial target with near to maximal effort using an actuated pocket knife blade [19]. The measured ranges were 2.6–9.2 m/s for velocities (mean 5.8 m/s), 12–104 Ns for momenta (mean 40 Ns) and 7–103 J (mean 36 J) for energies. Also, the study detected differences in the maximum measured force values depending on the stabbing technique, e.g., higher cutting forces in overhand stabs compared to short forward thrusts and horizontal style sweeps. Hence, the minimum momentum measured here to achieve a full-width blade penetration was slightly lower compared to the minimum momentum reached in planned near to maximal effort stabs determined in the aforementioned study.

The experimental findings highlight the challenge of applying subjective force scales such as “mild”, “moderate” and “severe” in regard to the degree of force, a concern previously noted using a different experimental setup [11]. For instance, the simple drop of the pendulum without manual acceleration, which might be interpreted as a “mild” force, and the additional moderate manual acceleration, which could be viewed as a “moderate” force, resulted in maximum force values exceeding those observed under high manual acceleration (“severe force”). Importantly, there are currently no established limits defining what constitutes mild or moderate from severe force, and these definitions would need to be specified for each individual stabbing instrument [4, 6]. The challenge of applying subjective force scales can only be addressed by providing experimental data at varying and quantified loads as done in this manuscript. Therefore, if the degree of force is referred to using a verbal rating scale, it should be accompanied by experimental data (ideally using the original weapon and varying impact velocities) and put into perspective in regard to the weapon (especially sharpness and shape of the blade) and the impacted anatomical region (especially the thickness of the impacted sample). If one of these factors is ignored by the expert witness or the factors are not considered in relation to each other, referring to force degrees remains an educated guess at best rather than an evidence-based statement. The difficulty in determining the degree of force using a verbal rating scale does not negate the value of biomechanical experiments in homicidal stab cases for court purposes. Firstly, the biomechanical experiments, which included three stabs into the contralateral temporo-parietal region of the victim using the original blade, indicated that a momentum of at least 10.1 Ns was necessary to achieve full-width blade penetration consistent with the homicide case. Secondly, all tests conducted with a momentum of at least 8.6 Ns resulted in either full-width blade penetration or fragmentation of the bone sample when samples of comparable thickness to the injured area in the homicide case were utilized. Lower momentums failed to achieve full-width blade penetration, despite the use of samples of comparable thickness to the injured region. This suggests that the momentum in the homicide case was likely higher than 10.1 Ns, which was insufficient to achieve full-width blade penetration in the conducted experiments. Notably, biomechanical investigations such as the one performed here can only determine the minimum degree of force necessary to cause an injury resembling that in a corresponding homicide case, rather than the actual degree of force applied. For instance, in the presented case, a minimum of 431 N was required for full-width blade entry into the bone. However, the actual stab force could have been higher. Therefore, it is more appropriate to refer to the “minimum degree of force” when assessing such cases or, preferably, using the momentum for comparison rather than force values. Furthermore, in cases of complete penetration or bone fragmentation, a lower reaction force is measured compared to when the blade abruptly stops within the bone. This is because the measured force results from the deceleration of the blade, which is significantly higher in case of low penetration depths and high impact velocities – as observed in Test 5. The measured force therefore depends on the mass of the arm and the knife, the type of fracture (slight or deep penetration, or bone fragmentation), which significantly influences the deceleration as well as the bone thickness, which determines the penetration depth.

In the future, biomechanical experiments to determine the minimum degree of force or momentum required to cause a specific injury should be increasingly advocated by forensic investigators. Police investigator attendance of the forensic autopsy is desirable in such cases to allow for fast an well-informed decision making processes, particularly in less frequent analyses such as biomechanical reconstructions. By compiling biomechanical studies that utilize various instruments and body regions, more objective data will become available for forensic pathologists to prepare for this question in court and provide more informed statements on the matter. Furthermore, this study demonstrates that, in particular cases, it is possible to conduct these experiments using the murder weapon and tissue from the victim, which significantly enhances the relevance of such investigations.

### Limitations

This study is limited by its sample size. Each stab, including the one in the homicide case, may have contributed to the dulling of the blade, potentially leading to increased forces required to produce the same injury with subsequent stabs [5]. Due to the limited frame rate and manual recording of the impact videos, only a restricted number of frames were available to determine the velocity just before the impact. Therefore, the velocities may have been subject to deviations when large motion increments between individual frames were present. The measured maximum forces from the second and third stabs on the victim’s bone sample may have been influenced by previous stabs to the same bone, possibly due to microfractures. Additionally, the necessary freeze-storage of the victim’s bone may have affected its microstructure, presumably resulting in lower force values compared to a scenario without a freeze-thaw cycle. Moreover, only skull samples with attached periosteum were tested [20]. In the homicide case, the knife penetrated several other tissue layers before reaching the outer table of the three-layered skull, and subsequently the inner table. However, the forces required to penetrate the soft tissues in this region, both inside and outside the skull, are considered negligible compared to those needed to completely perforate the skull [12, 13].

## Conclusions

By utilizing the victim’s tissue and the murder weapon for biomechanical analysis of the stabbing intensity in homicidal stab cases, the experimental setup closely replicates the conditions of the actual case. Forensic investigators should proactively recommend such biomechanical analyses and secure appropriate tissue samples during autopsy to gather objective experimental data that is pertinent to jurisdictional questions.

## Data Availability

The data is available on request.
